# Perceived stress and coping among adults in Germany: results from the ‘Health in Germany’ panel 2024

**DOI:** 10.25646/14260

**Published:** 2026-06-17

**Authors:** Christina Kersjes, Diana Peitz, Vera Birgel, Benjamin Wachtler, Caroline Cohrdes

**Affiliations:** Robert Koch Institute, Department of Epidemiology and Health Monitoring, Berlin, Germany

**Keywords:** Adults, Stress, Coping, Mental health, Prevalence, Education, Panel, Germany, Perceived Stress Scale, Short Adult Coping Scale

## Abstract

**Background:**

Stress and coping are key determinants of mental health. However, representative data for Germany remain scarce. This study provides population-based findings on the prevalence of perceived stress and coping as well as on their association.

**Methods:**

Data were derived from the representative study series ‘Health in Germany’ conducted by the Robert Koch Institute (wave 2024; n = 27,102; 51.1 % women; age range: 18 – 99 years). Descriptive analyses of stress (*Perceived Stress Scale*; PSS-10) and coping (*Short Adult Coping Scale*; *SACS-16*) were conducted by gender, age, and education, as well as regression analyses examining their associations.

**Results:**

Approximately 20 % of respondents showed elevated stress levels, particularly women, individuals of working age, and those with low or medium formal education. *Problem solving*, *proactive coping*, and *coping flexibility* were associated with lower perceived stress, whereas *repression* and *wishful thinking* were associated with higher perceived stress. Differences in coping strategies were observed primarily across age groups.

**Conclusions:**

The findings underscore the public health relevance of stress and indicate potential approaches for preventive measures by promoting suitable and target-group-specific coping strategies.

## 1. Introduction

Stress is an important risk factor for physical and mental health. Chronically perceived stress is considered particularly relevant to health, as it is more likely to be associated with long-lasting or even permanent changes in emotional, physical, and behavioral responses. These can influence both susceptibility to and the course of diseases [1–4]. Accordingly, stress is associated with both physical conditions (e.g., cardiovascular diseases) and mental health conditions (e.g., depression) [1, 5, 6], as well as with higher mortality [7] and lower mental well-being [8].

When a person experiences a situation as stressful (i.e., as significant and difficult to cope with), they subsequently apply cognitive, emotional, and behavioral coping strategies [9] (Infobox 1). The coping strategies used play a key role in how efficiently and quickly perceived stress can be managed [10, 11]. The relationship between stress and coping is reciprocal: stress initiates coping efforts, whose quality and fit are in turn associated with the extent of perceived stress [9].

**Key messages**

► Stress and coping are important indicators for mental health surveillance.
► Approximately 20 % of respondents reported elevated stress levels, particularly women, individuals of working age, and people with low or medium formal education.
► *Problem solving* and *coping flexibility* were frequently reported and were associated with lower stress; *repression* and *wishful thinking* were reported less often and were associated with higher stress.
► Depending on age, gender, and formal education, different coping strategies were more or less strongly associated with stress.
► In addition to structural prevention, behavioral stress prevention should be strengthened by promoting suitable coping strategies.

Consequently, perceived stress and coping are important factors associated with mental health, whose levels depend on both individual resources and the surrounding environment. Depending on life circumstances, events may be perceived as more or less stressful and different coping strategies may be applied [20]. Stress and coping may differ by age, gender, and socioeconomic position [21–23]. Stress in particular is therefore already included in surveillance systems in many countries (Canada [24, 25], UK [26], USA [27], Australia [28], New Zealand [29]). For Germany, however, apart from the irregular stress study conducted by Techniker health insurance (TK) with 1,000 – 1,400 representatively selected respondents [30, 31], no further regular representative surveys on stress or coping have so far been identified.

Coping is an approach for individual-centered measures within the framework of behavioral prevention, for example in the promotion of life skills in the school setting in the context of the health goal ‘Growing up healthy’ [32], and is increasingly the subject of public health strategies and recommendations in societal crises, such as the COVID-19 pandemic. Promoting coping strategies (e.g., maintaining social contacts and routines, acceptance of unpleasant feelings) was an important recommendation for managing multiple stressors during the COVID-19 pandemic [33, 34]. As an indicator, coping has so far been used in surveillance in only a few countries, such as Canada [24, 35]. This may be due to the complexity of the construct, the large number of operationalizations or length of assessment instruments [36]. For this reason, a brief coping instrument for population-based studies was developed and validated at the Robert Koch Institute (RKI), based on existing instruments identified in a review [36].

**Infobox 1 Stress and coping – theoretical background**

**Transactional stress model**

According to the transactional stress model by Lazarus and Folkman [9] stress arises when demands are perceived as significant (primary appraisal) and, at the same time, as difficult to cope with (secondary appraisal).

**Coping strategies**

Coping strategies serve to regulate the emotional stress associated with a situation and/or to manage the problem causing the stress [12]. The goals of coping may be both immediate (e.g., regulation of biophysiological stress responses) and intermediate in nature (e.g., socially normative functioning) [13].

**Adaptive and maladaptive coping strategies**

Coping strategies can be differentiated according to their effects on mental health. Coping strategies that attempt to actively influence the situation (e.g., problem solving, planning, or instrumental action) are often considered adaptive (i.e., helpful) [10, 14] and are associated with better mental health [15]. Strategies that are more oriented toward disengaging from the situation (e.g., avoidance or repeated postponement) or that place an excessive focus on the stressor without resolving it (e.g., catastrophizing) are often considered maladaptive (i.e., harmful) [16] and are frequently associated with poorer mental health [17]. However, whether coping is adaptive or maladaptive depends on its definition and on the context in which it takes effect [10]. Thus, maladaptive coping may also have positive effects in the short term, but may lead to higher perceived burden in the long term [18].

**Coping flexibility**

Flexible coping, i.e., the ability to vary strategies in a situation-appropriate manner, is associated with lower perceived stress and better mental health [12, 19].

In Germany, the Mental Health Surveillance (MHS) was established at the RKI in 2019, with the aim of continuously reporting systematically selected core indicators of mental health in the general population [37]. Since stress-related diseases are widespread, have substantial effects on quality of life, and are associated with high economic costs [38], it is of central importance to assess both stress and coping strategies in the MHS. Corresponding data can provide indications of risk groups and help develop preventive measures that specifically promote the ability to cope with stress in different population groups. The newly established RKI panel ‘Health in Germany’ offers the opportunity to quantify these indicators using population-based survey data both cross-sectionally and longitudinally, and to examine them in relation to other determinants and outcomes of mental health [39]. To establish both indicators in the regular reporting of the MHS, the present study therefore has the following aims:

► To provide current findings on perceived stress in the population in Germany, stratified by gender, age, and formal education;
► To provide current findings on different coping strategies reported in the population in Germany, stratified by gender, age, and formal education;
► To examine the association between perceived stress and coping, as well as differences in these associations across age, gender, and education groups.

## 2. Methods

### 2.1 Study design and sample

The data come from the first annual wave of the study series ‘Health in Germany’, established at the RKI in 2024. The sample is based on a two-stage stratified random selection from 359 municipalities and address registers of the residents’ registration offices (Infobox). Invited individuals could participate either online or by paper-and-pencil postal questionnaire. The panel comprises 47,676 registered participants aged 18 years and older, 53 % of whom are female. In 2024, three survey time points were conducted, with four thematically varying questionnaire variants covering, among other topics, physical and mental health, their determinants, and health care utilization [40]. For this purpose, in a predefined rotating procedure, participants received one of the four questionnaire variants in each of the three sub-waves. In the first sub-wave, additional in-depth questions on sociodemographic information were collected. Indicators of mental health were assessed in questionnaire C (Infobox). Data collection took place from May 2024 to January 2025; the response rate ranged between 75 % and 81 %, depending on the sub-wave. A detailed description of the methodology and response rate (including stratification by age and gender) can be found elsewhere [41].

### 2.2 Variables

#### Perceived stress

To assess stress, the German version [42] of the widely used *Perceived Stress Scale-10* (PSS-10) [43] was used. The PSS is conceptually based on the transactional stress model [44] and assesses the extent to which individuals perceive their lives as unpredictable, uncontrollable, and overwhelming [43]. Recent studies indicate a bifactorial structure of the PSS-10, consisting of a general factor and two specific subfactors: *perceived helplessness* and *perceived self-efficacy* [42]. *Perceived helplessness* reflects primary appraisal in the transactional stress model, i.e., the individual’s response to stress, whereas *perceived self-efficacy* reflects secondary appraisal, i.e., the subjectively perceived ability to cope with stress [45]. Using ten items, the PSS-10 assesses on a 5-point Likert scale (0 = *never* to 4 = *very often*; range 0 – 40) how often individuals have thought or felt in a certain way during the past month. The negatively worded items 1, 2, 3, 6, 9, and 10 form the factor *perceived helplessness* (range: 0 – 24), while the positively worded items 4, 5, 7, and 8 (reverse-coded) form the factor *perceived self-efficacy* (range: 0 – 16). Higher values reflect higher perceived stress. For surveillance purposes, a risk group for elevated stress levels was additionally defined. Given the two-factorial structure of the PSS-10 [42, 45] this was operationalized as values at or above the 75th percentile [cf. 46] on both factors.

**RKI Panel ‘Health in Germany’ 2024**

**Data holder:** Robert Koch Institute

**Objectives:** To provide comprehensive data on the health status, health-related behaviour and health care of the population in Germany, with the future possibility of longitudinal comparisons and analysis of trends over time

**Study design:** Panel study with a mixed-mode approach (online and written-postal participation)

**Population:** German-speaking population aged 18 and over in private households with main residence in Germany

**Sample:** Probabilistic/representative sample of the ‘Health in Germany’ panel infrastructure

**Participants in the 2024 annual wave:** A total of 41,376 of the persons registered in the panel took part in at least one of the three sub-waves in 2024.

Questionnaire A: 14,759 women, 12,374 men, 66 persons with other gender identities
Questionnaire B: 14,828 women, 12,258 men, 61 persons with other gender identities
Questionnaire C: 14,709 women, 12,329 men, 64 persons with other gender identities
Questionnaire D: 14,872 women, 12,368 men, 66 persons with other gender identities

**Data collection:**

1st sub-wave: 28.05.2024 – 05.08.2024
2nd sub-wave: 12.08.2024 – 14.10.2024
3rd sub-wave: 28.10.2024 – 06.01.2025

More information at www.rki.de/panel-en

#### Coping

To assess coping strategies, the *Short Adult Coping Scale-16* (SACS-16) [36] was used. With two items per scale, it assesses eight central coping strategies (*emotional support*, *instrumental support*, *perseverance*, *coping flexibility*, *problem solving*, *repression*, *wishful thinking*, and *proactive coping*). Participants were asked to rate statements according to what they had thought, felt or done in difficult and significant situations in the past, using a 4-point Likert scale ranging from 0 = *strongly disagree* to 3 = *fully agree*. Model fit and measurement invariance have been demonstrated for the SACS-16 [36]. A description of the coping strategies assessed in the SACS is provided in Table 1.

As the short versions of the PSS-10 (PSS-2 & 2 [52]) and the SACS-16 (SACS-8 [36]) are intended to be used for future surveillance purposes at the RKI, the present study included corresponding testing and validation of both the long and short instruments. Details on the PSS-2 & 2 and the SACS-8 can be found in the Supplementary material under ‘Additional information on 2.2 Variables’.

#### Sociodemographic variables

Gender identity was used to describe gender differences, and the categories female and male were analyzed. Persons with a diverse gender identity were not reported due to small case numbers. Participants’ age was categorized into the groups 18 – 29, 30 – 44, 45 – 64, 65 – 79, and 80 – 99 years. To assess formal education, the highest school or vocational qualification was recorded according to the 2011 version of the International Standard Classification of Education (ISCED)-Scheme [53] and classified into the categories low (lower secondary education or below, ISCED levels 0 – 2), medium (upper secondary or post-secondary education, ISCED levels 3 – 4), and high (tertiary education, ISCED levels 5 – 8).

### 2.3 Statistical methods

All analyses were conducted using R version 4.4.0 and RStudio version 2025.05.1 + 513, based on dataset version 5 of the RKI panel.

To address the planned research questions, it was necessary that the employed instruments reliably and validly assess the underlying constructs. For this purpose, procedures for examining the factor structure and measurement invariance analyses were conducted. A comprehensive presentation of this instrument testing would exceed the scope of this article. Details on the methodological approach are provided in the Supplementary material under ‘Additional information on 2.3 Statistical methods – instrument testing’.

In a first step, descriptive statistics were calculated for the variable *perceived stress* (PSS-10) (comprising four outcomes: the total score, the subscale score for *perceived helplessness*, the subscale score for *perceived self-efficacy*, and exceeding the 75th percentile on both factors (risk group)) as well as for *coping* (SACS-16) stratified by gender, age group, and education. Further analyses were conducted separately for women and men.

To examine group differences (by age group, gender, and formal education) in *perceived stress* and in the reporting of coping strategies, as well as associations between *stress* and *coping*, linear regression models were calculated for the metric stress outcomes (total score, subscale score for *perceived self-efficacy*, and for *perceived helplessness*) and for coping strategies. A logistic regression model was calculated for the risk group. All regression models included coping strategies and sociodemographic factors (gender, age, education) as predictors. Furthermore, interactions between coping and sociodemographic variables were included in the models to identify potential moderating effects. Significant interactions were subsequently examined with regard to the slope of the association for different levels of the moderator (‘simple slope analysis’). The *interactions* package was used to visualize and interpret the nature and strength of the interactions between variables.

For all descriptive and association analyses, a population weight was applied, implemented in R using the *srvyr* package in order to ensure representative estimates for the total population [57].

## 3. Results

The sample for questionnaire C comprised 27,102 individuals aged 18 – 99 years (51.1 % women). Further sample characteristics are shown in Table 2.

The results of the instrument testing are provided in the Supplementary material (‘Additional information on 3. Results – instrument testing’ and Tables 1 – 10).

### 3.1 Perceived stress

Stratified mean values for perceived stress as well as percentages of persons with elevated stress levels are shown in Table 3. Corresponding values stratified by women and men are provided in the Supplementary material (Table 11 ). Approximately 20 % of the sample showed elevated stress levels, i.e., their stress levels were above the 75th percentile of the distribution on both factors (*perceived helplessness* and *perceived self-efficacy*). These were predominantly women, persons aged 18 – 64 years, and persons with low or medium formal education.

Across all stress outcomes considered, the following differences with small to medium effect sizes [58] were observed (see Supplementary material Table 12): women reported significantly higher perceived stress than men. Young persons aged 18 – 29 years reported the highest perceived stress. With increasing age, lower perceived stress was reported up to the age group 65 – 79 years. In the oldest age group (80 – 99 years), perceived stress was again somewhat higher, but still significantly lower compared with young adults. Differences by education were also observed, with persons in the high education group reporting less stress. The differences found by age and education applied almost entirely when women and men were analyzed separately. Only in some cases did not all age group comparisons reach significance. Among men, this concerned the comparison between 18- to 29-year-olds and 30- to 44-year-olds for *perceived helplessness* and for those with elevated stress levels.

### 3.2 Coping

Stratified mean values for the coping strategies assessed using the SACS-16 are shown in Table 4 (for women and men separately, see Supplementary material Table 13). The most frequently reported coping strategies were *problem solving*, *perseverance*, and *coping flexibility*. The least frequently reported strategies were *repression*, *wishful thinking*, and *proactive coping*. Across the coping strategies, the following differences with small to medium effect sizes [58] were observed (Supplementary material Table 14): women reported *emotional support*, *instrumental support*, *perseverance*, *repression*, and *wishful thinking* more often than men, and reported *problem solving* and *proactive coping* less often. The higher the age, the less frequently the coping strategies *emotional support*, *instrumental support*, *repression*, *wishful thinking*, and *proactive coping* were reported. By contrast, the coping strategies *coping flexibility*, *problem solving*, and *perseverance* were reported more frequently up to the age group of 65- to 79-year-olds. Persons aged 80 years and older, however, again reported lower levels of *coping flexibility*, *problem solving*, and *perseverance*. In this age group, these strategies were at a similar or lower level than in the youngest age group. The higher the level of formal education, the more frequently *emotional support*, *instrumental support*, *perseverance*, *coping flexibility*, *problem solving*, and *proactive coping* were reported, and the less frequently *repression* and *wishful thinking* were reported. The age- and education-related differences were also found separately for women and men, with few exceptions (men: *perseverance* and *repression* in 18- to 29-year-olds vs. 30- to 44-year-olds not significant; women: *coping flexibility* in 18- to 29-year-olds vs. 80- to 99-year-olds not significant).

### 3.3 Association between perceived stress and coping

Associations between *stress* and *coping*, adjusted for gender, age, and education, are shown in Table 5. The coping strategies *coping flexibility*, *problem solving*, and *proactive coping* were significantly associated with lower perceived stress, whereas *repression* and *wishful thinking* were associated with higher perceived stress. Higher *emotional support* was significantly positively associated only with the factor *perceived helplessness*, but not with *perceived self-efficacy*. No significant association was found between *stress* and *instrumental support* or *perseverance*.

To examine whether the associations between *coping* and *stress* varied by gender, age, and/or education, a regression model with gender, age, and education groups as categorical moderators was estimated (Supplementary material, Table 15). The interaction terms were interpreted as differences in the associations compared with the respective reference group (male, 18- to 29-year-olds, and low education, respectively). Simple slope analyses were additionally used to describe the direction and strength of the group-specific associations (Supplementary material, Table 16). The moderation findings are summarized according to their consistency across the four stress outcomes. Interactions that were significant for at least three outcomes are reported as consistent indications, whereas interactions for one or two outcomes are reported as outcome-specific findings.

Consistent indications of moderation were observed primarily for age differences. The association between *coping flexibility* and *perceived stress* varied significantly by age group: associations were stronger particularly among persons aged 30 – 44 years and 45 – 64 years than among those aged 18 – 29 years. A comparable consistent pattern was observed for *problem solving* among persons aged 45 – 64 years: they showed stronger associations with *perceived stress* than the youngest age group. In addition, there was a consistent indication of moderation by level of education for *perseverance*, with stronger associations for the medium compared with the low education group.

Further significant interactions were limited to individual stress outcomes. These can be found in the Supplementary material (Tables 15 and 16); their interpretation is provided in the discussion.

## 4. Discussion

### 4.1 Summary

Almost 20 % of participants showed elevated stress levels, particularly women, persons of working age, and persons with low or medium levels of formal education. The most frequently reported coping strategies included active coping strategies (*problem solving*, *perseverance*) and *coping flexibility*, whereas *repression*, *wishful thinking*, and *proactive coping* were reported least frequently. The results of the analysis of associations between *stress* and *coping* showed that *coping flexibility*, *problem solving*, and *proactive coping* were significantly associated with lower perceived stress, while *repression* and *wishful thinking* were associated with higher perceived stress. Both the reporting of individual coping strategies and their association with stress differed by gender, age, and formal education.

### 4.2 Interpretation and implications

#### General interpretation

The sample of the present study showed higher stress levels (PSS-10 total score in the range of 17.6 – 12.7 for persons aged 18 – 99 years) compared with the norm values for the general population in Germany collected in 2014 (PSS-10 total score in the range of 14.1 – 11.9 for persons aged 14 – 95 years) [45]. An increase in perceived stress over the past decade has been found both in international studies [59, 60] and in studies for Germany [30, 31]. According to the TK study, the proportion of people in Germany who often or sometimes feel stressed increased from 57 % in 2013 to 66 % in 2025 [31]. Possible reasons have so far been scarcely investigated; in some cases, precarious labor market conditions, political instability, or lower social cohesion have been discussed [59]. In recent years, population-level stressors such as the COVID-19 pandemic, Russia’s war on Ukraine and its economic consequences, such as inflation, as well as concerns about climate change, have been added and may have contributed to a further increase in perceived stress. Accordingly, in 2021, 47 % of respondents in the TK study reported that their perceived stress levels had increased since the beginning of the pandemic [30] and in 2025, 53 % reported feeling burdened by political and societal problems such as wars [31]. In the context of these events, other indicators of mental health, such as depressive symptoms, anxiety symptoms, and self-rated mental health, have also shown a deterioration [61–63]. Nevertheless, it should be emphasized that our findings indicate low to moderate average perceived stress levels (PSS-10 total mean score of 15.2, with a range of 0 – 40), rather than high levels of stress across the whole population.

With the present publication, population-based results on the SACS-16 can be presented for the first time.

#### Gender-specific differences in *perceived stress* and *coping*

The finding that women report higher perceived stress has been widely replicated across countries [e.g. 5, 23, 45, 60]. The measurement invariance analyses show that the measurement of stress is comparable for men and women. This suggests that the observed gender differences are not primarily attributable to methodological bias. Possible reasons may be that women are more often exposed to different stressors, interpret stress differently, or differ in their coping strategies [64]. For example, women experience more daily minor stressors and more chronic stress [64, 65], and perceive major life events as less controllable than men [64]. The latter is consistent with the significant gender differences we found on the dimension *perceived helplessness*.

We also found differences between women and men in coping strategies. Empirical studies show that women more often report social support, emotion-based, and avoidant coping strategies [64, 66, 67], whereas men more often report active problem-focused and rational coping strategies [64, 66]. Differences in socialization, among other factors, are cited as a possible explanation. Different expectations regarding gender roles, with men expected to be independent and rational and women expected to be emotional and supportive, may contribute to such gender-specific coping strategies [64, 66]. The present study is consistent with previous findings, with rather small effect sizes for gender differences in problem-focused and avoidant strategies compared with *emotional support* and *instrumental support*.

The association between lower *stress* and *problem solving* was particularly pronounced among men compared with women. The coping strategy *emotional support* was also differently associated with *stress* in women and men. While *emotional support* tended to be associated with stress among men, this association was less pronounced among women. This can be interpreted in the context of gender-specific social role expectations, although other reasons, such as different needs or behaviors in the context of psychobiological stress responses [68], may also be relevant.

#### Age-specific differences in *perceived stress* and *coping*

Our study finding that stress was highest in young adulthood and decreased thereafter is consistent with evidence from cross-sectional and longitudinal studies [69]. The following reasons may have contributed to this:

- In the literature, it is assumed that the type and number of everyday stressors change over the life course, among other things due to consolidating social roles and changing developmental tasks [69, 70]. However, the definition of the risk group indicated that persons up to 64 years of age had the greatest risk of elevated stress levels on both factors of the PSS-10. This may be due to multiple burdens, for example from work, care work for children and ageing parents, and financial responsibility [71]. This finding supports the results of the TK study, according to which stress only becomes significantly less important after entry into retirement age [30].
- In addition, numerous findings support a so-called positivity effect, i.e., with increasing age, attention is increasingly directed toward positive aspects [72]. This may be associated with older persons seeking out stressful situations less often or perceiving them as less stressful [69].
- Coping strategies also appear to be increasingly developed over the life course [21]. Consistent with this, the present study found that *coping flexibility* (i.e., the ability to draw on a range of acquired strategies) and *problem solving* were reported more frequently the older the age group – except for the oldest-old. Aldwin et al. [73], found that older persons used coping strategies more effectively and, for instance, used avoidant coping strategies less often. This is consistent with the present findings, which showed that the older the age group, the less frequently maladaptive strategies such as *repression* and *wishful thinking* were reported.

In the age group 80–99 years, perceived stress increased again in our sample. Many other studies group older people together, for example from retirement age onward, which makes it difficult to contextualize perceived stress specifically in this age group. In a population-based study, Osmanovic et al. [74] divided persons aged 66 – 97 years into three age groups and, similarly to the present study, found an increase in stress levels, with the highest values observed in the age group over 81 years. Possible reasons may be that the number of stressors increases again, for example due to health-related burdens, functional limitations, and experiences of loss [70], that positive emotional experiences no longer increase or even decrease again (stagnation or reversal of the positivity effect) [72], and that the experienced intensity of stressors increases [70]. The latter may also be due to fewer coping resources [70]. Our data also point in this direction, as *perseverance*, *coping flexibility*, and *problem solving* decreased again in the oldest age group.

Our findings also showed particularly strong associations between individual coping strategies and lower stress in certain age groups. For *proactive coping*, we found a particularly strong association among young adults (18 – 29 years), for *coping flexibility* among 30- to 64-year-olds, and for *problem solving* among 45- to 64-year-olds. These findings point to target-group-specific potential of selected coping strategies. The association between *instrumental support* and lower *stress* was particularly pronounced in the age group 30 – 44 years but reversed from the age group 65 years and older onward, such that *instrumental support* was associated with lower *perceived self-efficacy* among 65- to 79-year-olds and with higher *perceived helplessness* among 80- to 99-year-olds.

#### Education-specific differences in *perceived stress* and *coping*

A high level of formal education was associated with lower *perceived stress*. A large proportion of persons with elevated stress levels had low or medium formal education. This is consistent with previous study findings [5, 45, 75]. The present study is the first to examine differences by education not only in the total score but also in the subfactors of the PSS-10. The findings suggest that education-related differences in this study were more strongly associated with the appraisal of one’s own possibilities for coping than with feelings of helplessness in stressful situations. In this appraisal, in addition to situational variables, existing resources for coping (psychological, social, and material) as well as coping strategies are taken into account [76, 77]. Our findings show that persons in the high education group reported more adaptive (e.g., *problem solving*) and fewer maladaptive coping strategies (e.g., *repression*). Also, many coping resources are socially unevenly distributed: for example, higher self-efficacy [78], sense of control and social support [79], as well as greater material resources [80], are associated with higher education. These differences in coping resources and strategies may be associated with differences in *perceived self-efficacy* in stressful situations [81]. Our findings indicate that persons in the high education group more frequently reported *emotional support* and *instrumental support*. This may be related to education-related differences in social support [79] which is an important factor influencing stress, coping, and mental health [5, 81]. The regression analysis provides indications that *instrumental support* may be a possible approach for interventions among persons with low educational attainment, as the association between *instrumental support* and lower *stress* was particularly strong in this group. Furthermore, coping resources may influence how controllable or manageable a problem is perceived to be. *Problem solving* is used particularly often when the situation is controllable [16]. *Perseverance* can become maladaptive when the goal is not manageable [47]. Education-related differences in coping resources may therefore have contributed to persons in the low education group reporting *problem solving* less frequently and to *perseverance* being associated with higher *perceived helplessness* in this group.

#### Implications

Prevention programs that address stress reduction are particularly relevant given the association between stress and mental disorders. In principle, both structural and behavioral prevention approaches may be considered. In the sense of proportional universalism [82], these measures can be directed universally at the entire population and, at the same time, proportionally more strongly at particularly burdened groups. Based on the present study, corresponding risk groups for elevated stress levels can be described: women, persons of working age, and persons with low or medium formal education.

An important setting in which prevention measures for persons of working age can be delivered is the workplace. The Stress Report of the Federal Institute for Occupational Safety and Health [83], which is representative of employees, shows that important work-related risk factors for mental strain include high work intensity, unfavourable working hours (e.g., more than 40 hours/week, insufficient rest periods, shift work, extended availability), and factors related to leadership and organization (e.g., low appreciation). They recommend measures that address structural working conditions, such as adequate staffing, compliance with minimum rest periods and avoidance of excessively long working hours, qualification of managers with regard to health-promoting leadership behavior, and consistent implementation of workplace health management and return-to-work management. Measures relating to job and income security are particularly important for younger persons and persons with low education, while measures to promote work-life balance are particularly important for women. At the individual level, behavioral prevention measures such as stress management training and individual counseling and support services are recommended [83].

At the level of behavioral prevention, our study findings indicate that measures to promote adaptive coping strategies and reduce maladaptive coping strategies may represent relevant approaches for stress prevention. These could, for example, teach strategies such as *problem solving* and *coping flexibility* while also taking into account target-group-specific coping strategies such as *proactive coping* for younger people. Suitable programs could be based on mindfulness or cognitive behavioral therapy. Both interventions lead to the use of more adaptive coping strategies [84, 85] and lower perceived stress [86, 87]. Although there are so far only few evaluated universal coping interventions available, examples show how the promotion of coping strategies can be successfully applied in primary prevention approaches [88]. In addition to setting-based approaches, for example in schools and workplaces, prevention courses offered by health insurance funds are also suitable in this context.

### 4.3 Limitations and outlook

One strength of the present study is the large number of participants in the ‘Health in Germany’ panel. Recruitment through residents’ registration offices and the use of a study-specific weighting factor achieve a high degree of representativeness for the adult residential population living in Germany. Nevertheless, certain biases, for example due to selective non-participation, cannot be ruled out. In addition, this study is based on a cross-sectional design. The observed associations between perceived stress and coping therefore cannot be clearly interpreted in terms of their direction. For example, it is equally plausible that higher stress promotes the reporting of maladaptive coping strategies, as it is that maladaptive coping contributes to higher stress.

The PSS-10 is a widely used and validated instrument for assessing stress and the definition of a risk group for elevated stress was introduced for the purpose of mental health surveillance, not for the diagnosis of clinical symptoms. When considering the mean value of the PSS-10, different groups were identified than when defining the risk group. This should be taken into account, particularly when developing public health measures; therefore, the additional presentation of the risk group appears useful in the further surveillance of this indicator.

The SACS-16 is a brief and validated instrument developed for population studies that assesses important dispositional coping strategies. When interpreting the findings, it should be taken into account that the scales showed partial limitations regarding comparability between groups and reliability, which need to be further monitored and examined for opportunities for optimization (Supplementary material ‘Additional information on 4. Discussion – instrument testing’).

Regarding the stratification variables, education does not sufficiently capture the influence of socioeconomic status on perceived stress and coping. More in-depth analyses should therefore also consider, for example, income and occupation, which have been shown to be associated with stress and coping [21, 45]. Future research could also examine specific populations, such as persons with a history of migration or persons in precarious employment, as these groups may be exposed to particularly high levels of stress and could benefit from specific coping strategies. Furthermore, coping strategies are supported by personal, material, and social resources in coping with stress, which may be socially unequally distributed [77]. To obtain a more comprehensive picture of coping with stress, future in-depth studies could additionally consider corresponding coping resources as well as coping behavior (e.g., substance use, relaxation techniques, gaming), specifically focusing on socioeconomic inequality in stress and coping and using suitable methods to explain it.

In order to better design public health interventions, the reasons for perceived stress as well as the differential effects of acute and chronic stress should also be assessed [89]. Further important insights may also be provided by studies within a salutogenic approach, i.e., examining the group of persons with low perceived stress and the resources associated with it. Likewise, the population-based variability or stability of coping strategies should be examined. This includes possible changes in times of crisis, as these are situations in which, for example, there may be less control over stressors, and some coping strategies may not be applicable. A study from the COVID-19 pandemic showed, for example, that meaning-focused coping strategies, such as positive reappraisal, were of high importance [90]. This variability highlights the potential of coping interventions that promote adaptive coping strategies and modify maladaptive strategies [91].

### 4.4 Conclusion

The present study was able to quantify perceived stress and coping strategies using a panel study representative of Germany. In view of globally increasing stress levels and the occurrence of multiple crises, both indicators may serve as important indicators for the surveillance of mental health in the population. The instruments used were found to be suitable for this purpose and may be used in future surveillance, including in their economical short forms, with continued methodological monitoring.

For the development of targeted prevention measures, we were able to identify women, persons of working age, and persons with low or medium formal education as relevant risk groups. At the same time, the findings point to potential approaches for behavioral prevention measures: in particular, active strategies such as *problem solving* and the ability to draw on different strategies in dealing with stress (*coping flexibility*) were associated with lower perceived stress.

In addition to structural prevention approaches, for example in the work context, measures to promote adaptive coping strategies could make an important contribution to the prevention of stress and stress-related consequences. As the relevance of individual coping strategies may vary by age, gender, and formal education (e.g., *instrumental support* among persons with low formal education), prevention and intervention programs can be designed in a target-group-specific manner and tested and implemented in different settings, such as educational, work, and household contexts, across the life span.

## Figures and Tables

**Table 1:** Explanations and example items for the eight coping strategies of the SACS-16. Sources: ^1^ [16], ^2^ [47], ^3^ [48], ^4^ [49], ^5^ [50], ^6^ [51]

Coping strategy	Explanation	Example item from the SACS-16
Emotional support	Seeking social support to obtain moral support, sympathy, or understanding^1^	I let my feelings out to a friend.
Instrumental support	Seeking social support to obtain advice, help, or information^1^	I let my friends help me.
Perseverance	Diligence and perseverance in pursuing a goal despite challenges or difficulties^2^	I stood firm and fought for what I wanted in the situation.
Coping flexibility	Situation-appropriate adjustment of coping strategies^3^	Even if the stressful situation has worsened, I could cope by using another strategy.
Problem solving	Actions intended to change the cause of stress^1^	I’ve been taking action to try to make the situation better.
Repression	Behavioral patterns and internal attitudes based on the avoidance of negative emotions^4^	I refused to believe it had happened.
Wishful thinking	Fantasizing about or hoping that the situation will improve without actively changing anything^5^	I fantasized about how things could have been different.
Proactive coping	Coping directed toward future, rather than current stressors and opportunities, for example by building resources, preventing problems, etc^6^	I liked seeking out challenges and was also willing to take risks.

**Table 2:** Sample description of the RKI Panel ‘Health in Germany’ 2024 – questionnaire C. Source: RKI Panel 2024

Characteristic		n	%
Total		27,102	100
Gender	Women	14,762	51.1
	Men	12,340	48.9
Age (in years)	18 – 29	3,829	15.9
	30 – 44	5,761	23.5
	45 – 64	9,025	33.4
	65 – 79	6,201	18.5
	80 – 99	2,286	8.7
Education	Low	5,201	33.3
	Medium	12,964	46.4
	High	8,888	20.3

n = unweighted number; % = weighted percentage; missing values for education: n = 49.

**Table 3:** Weighted stratified mean values for *perceived stress* and proportion of persons with elevated perceived stress based on the PSS-10 (n = 27,102). Source: RKI Panel 2024

Characteristic		Total score		*Perceived helplessness*		*Perceived self-efficacy*		*%* with elevated perceived stress (≥ 75th percentile)	
		M	95 % CI	M	95 % CI	M	95 % CI	*%*	95 % CI
Total		15.2	[15.0 – 15.3]	9.1	[9.0 – 9.2]	6.1	[6.0 – 6.1]	19.9	[19.2 – 20.7]
Gender	Women	15.9	[15.7 – 16.1]	9.6	[9.5 – 9.8]	6.2	[6.2 – 6.3]	11.5	[10.8 – 12.2]
	Men	14.4	[14.2 – 14.6]	8.5	[8.4 – 8.6]	5.9	[5.8 – 6.0]	8.4	[7.8 – 9.1]
Age (in years)	18 – 29	17.6	[17.3 – 17.9]	10.7	[10.5 – 10.9]	6.9	[6.8 – 7.1]	4.4	[4.0 – 4.9]
	30 – 44	16.2	[16.0 – 16.5]	9.9	[9.7 – 10.0]	6.4	[6.3 – 6.5]	5.7	[5.2 – 6.2]
	45 – 64	14.6	[14.4 – 14.8]	8.8	[8.7 – 9.0]	5.8	[5.7 – 5.9]	6.5	[6.0 – 6.9]
	65 – 79	12.7	[12.4 – 12.9]	7.4	[7.2 – 7.5]	5.3	[5.2 – 5.4]	1.9	[1.7 – 2.2]
	80 +	15.1	[14.7 – 15.5]	8.6	[8.3 – 8.9]	6.5	[6.3 – 6.6]	1.4	[1.2 – 1.6]
Education	Low	15.8	[15.5 – 16.0]	9.1	[8.9 – 9.3]	6.7	[6.6 – 6.8]	7.1	[6.3 – 7.8]
	Medium	15.2	[15.1 – 15.4]	9.2	[9.1 – 9.3]	6.0	[5.9 – 6.0]	9.7	[9.1 – 10.3]
	High	14.0	[13.8 – 14.2]	8.7	[8.6 – 8.8]	5.3	[5.2 – 5.4]	3.2	[2.9 – 3.5]

M = mean; CI = confidence interval; range for the total score: 0 – 40; range for *perceived helplessness*: 0 – 24; range for *perceived self-efficacy*: 0 – 16

**Table 4:** Weighted stratified mean values for *coping* based on the SACS-16 (n = 27,102). Source: RKI Panel 2024

Characteristic		*Emotional support*		*Instrumental support*		*Perseverance*		*Coping flexibility*		*Problem solving*		*Repression*		*Wishful thinking*		*Proactive coping*	
		M	95% CI	M	95% CI	M	95% CI	M	95% CI	M	95% CI	M	95% CI	M	95% CI	M	95% CI
Total		2.8	[2.8–2.8]	2.6	[2.6–2.7]	3.1	[3.1–3.1]	3.0	[3.0–3.0]	3.2	[3.2–3.2]	1.9	[1.9–1.9]	2.2	[2.1–2.2]	2.2	[2.1–2.2]
Gender	Women	3.0	[3.0–3.1]	2.8	[2.7–2.8]	3.1	[3.1–3.2]	3.0	[3.0–3.0]	3.2	[3.1–3.2]	2.0	[1.9–2.0]	2.2	[2.2–2.2]	2.1	[2.1–2.1]
	Men	2.6	[2.6–2.6]	2.5	[2.5–2.5]	3.1	[3.0–3.1]	3.0	[3.0–3.0]	3.2	[3.2–3.2]	1.9	[1.9–1.9]	2.1	[2.1–2.1]	2.2	[2.2–2.2]
Age (in years)	18–29	3.0	[2.9–3.0]	2.8	[2.7–2.8]	3.1	[3.0–3.1]	2.8	[2.7–2.8]	3.0	[3.0–3.1]	2.1	[2.0–2.1]	2.6	[2.6–2.6]	2.3	[2.3–2.3]
	30–44	3.0	[2.9–3.0]	2.7	[2.7–2.8]	3.1	[3.1–3.2]	2.9	[2.9–3.0]	3.2	[3.2–3.2]	2.0	[2.0–2.0]	2.3	[2.3–2.4]	2.3	[2.2–2.3]
	45–64	2.9	[2.9–2.9]	2.7	[2.7–2.7]	3.2	[3.2–3.2]	3.0	[3.0–3.1]	3.3	[3.3–3.3]	1.9	[1.9–1.9]	2.1	[2.0–2.1]	2.1	[2.1–2.2]
	65–79	2.6	[2.6–2.7]	2.5	[2.5–2.5]	3.1	[3.1–3.1]	3.1	[3.1–3.2]	3.3	[3.3–3.3]	1.8	[1.8–1.8]	1.9	[1.8–1.9]	2.1	[2.1–2.1]
	80 +	2.3	[2.3–2.4]	2.3	[2.2–2.3]	2.8	[2.7–2.8]	2.8	[2.8–2.9]	2.9	[2.9–3.0]	1.8	[1.8–1.8]	1.9	[1.8–1.9]	1.9	[1.9–2.0]
Education	Low	2.6	[2.6–2.6]	2.5	[2.5–2.5]	3.0	[2.9–3.0]	2.9	[2.9–2.9]	3.1	[3.0–3.1]	2.0	[1.9–2.0]	2.2	[2.1–2.2]	2.1	[2.1–2.1]
	Medium	2.9	[2.9–2.9]	2.7	[2.7–2.7]	3.1	[3.1–3.2]	3.0	[3.0–3.0]	3.2	[3.2–3.2]	2.0	[1.9–2.0]	2.2	[2.2–2.2]	2.2	[2.1–2.2]
	High	3.0	[3.0–3.1]	2.7	[2.7–2.8]	3.2	[3.2–3.2]	3.1	[3.1–3.1]	3.4	[3.3–3.4]	1.8	[1.8–1.8]	2.1	[2.0–2.1]	2.3	[2.3–2.3]

M=mean; CI = confidence interval; Range for each coping scale: 0–6

**Table 5:** Adjusted associations between *perceived stress* (PSS-10) and *coping* (SACS-16). Source: RKI Panel 2024

Predictor	Category	Total score		*Perceived helplessness*		*Perceived self-efficacy*		% with elevated perceived stress (≥ 75th percentile)	
		β	SE	β	SE	β	SE	AOR	95 % CI
Coping strategy	*Emotional support*	0.078	0.315	0.094^*^	0.226	0.032	0.176	1.499	[0.987 – 2.277]
	*Instrumental support*	- 0.046	0.312	- 0.058	0.236	- 0.016	0.165	0.798	[0.549–1.160]
	*Perseverance*	- 0.001	0.343	0.035	0.266	- 0.058	0.176	1.220	[0.765–1.945]
	*Coping flexibility*	- 0.135^***^	0.374	- 0.134^**^	0.286	- 0.104^*^	0.188	0.670	[0.435–1.033]
	*Problem solving*	- 0.137^***^	0.375	- 0.104^*^	0.293	- 0.154^**^	0.207	0.436^***^	[0.276 – 0.689]
	*Repression*	0.213^***^	0.284	0.199^***^	0.230	0.174^***^	0.159	1.680^***^	[1.226 – 2.301]
	*Wishful thinking*	0.282^***^	0.270	0.297^***^	0.203	0.178^***^	0.134	2.514^***^	[1.735 – 3.643]
	*Proactive coping*	- 0.077^*^	0.298	- 0.037	0.221	- 0.114^**^	0.152	0.606^*^	[0.408 – 0.900]

Note: reduced presentation; Table 15 in the Supplementary material shows the full table including interaction terms and the covariates age, gender, and education:

β = standardized regression coefficient; SE = standard error; AOR = adjusted odds ratio; CI = confidence interval; bold = statistically significant results

*p < 0.05

**p < 0.01

***p < 0.001
